# THE EFFECT OF GUARANTEED BASIC INCOME ON HOUSING ACCESS AND HEALTH AMONG OLDER PEOPLE EXPERIENCING HOMELESSNESS

**DOI:** 10.1093/geroni/igae098.0291

**Published:** 2024-12-31

**Authors:** Anthony Traver, Katie Calhoun

**Affiliations:** The Ohio State University, Columbus, Ohio, United States; The Ohio State University, Columbus, Ohio, United States

## Abstract

The confluence of population aging and rising housing costs across the United States has resulted in an unprecedented number of older people experiencing homelessness (OPEH). Worldwide, guaranteed basic income programs are being explored as strategies to prevent or end an episode of homelessness. The Denver Basic Income Project (DBIP), which began in 2022, provided a guaranteed income to adults experiencing homelessness in Denver, CO. DBIP used a randomized controlled design to compare the impact of two payment methods, monthly ($1,000 per month), and lump sum ($6,500 the first month, $500 per month after that), to an active control group ($50 per month). This study used a subsample of DBIP participants aged 50 and older at the time of enrollment (n=221) to analyze the 12-month impact of guaranteed income on housing access, overall health (measured by the SF-36), and emergency department visits among OPEH in Denver, CO. Generalized linear mixed-effects models were used to estimate the effect of intention to treat on outcomes of interest. The increase in the likelihood of obtaining housing was significantly larger for the monthly and lump sum groups than the control group. Group assignment was not associated with a difference in the likelihood of visiting an emergency department or a change in overall health. Results indicate that guaranteed income programs can support housing access among OPEH but may not alter health status over twelve months.

